# The NT-ProBNP Test in Subjects with End-Stage Renal Disease on Hemodialysis Presenting with Acute Dyspnea: Is Knowing Worth the Cost?

**DOI:** 10.1155/2013/836497

**Published:** 2013-03-07

**Authors:** Shaffer R. S. Mok, Jose Avila, Barry Milcarek, Richard Kasama

**Affiliations:** ^1^Division of Internal Medicine, Department of Medicine, Cooper University Hospital, 1 Cooper Plaza, 401 Haddon Avenue, 3rd Floor, Camden, NJ 08103, USA; ^2^Division of Nephrology, Department of Internal Medicine, Cooper University Hospital, 1 Cooper Plaza, 401 Haddon Avenue, 3rd Floor, Camden, NJ 08103, USA

## Abstract

*Background*. The NT-ProBNP/BNP test has been validated as a marker for determining the etiology of acute dyspnea. In the setting of end-stage renal disease on hemodialysis (ESRD on HD), the utility of the NT-ProBNP/BNP test has not been validated. This study examines the clinical utility of the NT-ProBNP test in the setting of ESRD on HD patients presenting with acute dyspnea. *Methods*. A retrospective case series of 250 subjects were admitted to Cooper University Hospital, 07/2010-03/2011, with ESRD and HD presenting with dyspnea. The incidences of echocardiography, cardiology consultation, and NT-ProBNP elevated and normal were examined. Correlation coefficients were calculated for NT-ProBNP with age (years), estimated dry weight (kg), amount of fluid removed (L), and ejection fraction (EF in %) among other echocardiography parameters. *Results*. Of the total sample 235 patients had NT-ProBNP levels performed. Cardiology consults were placed in 68.8% and 58% who underwent echocardiography. Of those for whom an echocardiography was performed estimated mean EFs of 54.6%, 50.8%, and 61.7% were observed among the NT-ProBNP elevated group, normal group, and no NT-ProBNP group, respectively. No differences were detected in all other echocardiography measurements. No correlation was observed between NT-ProBNP and age (*r* = 0.05), baseline EDW (*r* = −0.09), amount of fluid removed (*r* = 0.07), or EF (*r* = 0.02). *Conclusion*. In the setting of ESRD on HD, the NT-ProBNP test has no clinical utility in determining the etiology of acute dyspnea. This can be demonstrated through echocardiographic and therapeutic parameters measured in this study.

## 1. Introduction

Both N-Terminus Pro Brain Natriuretic Peptide (NT-ProBNP) and Brain Natriuretic Peptide (BNP) hormones have been used in patients presenting with a chief complaint of dyspnea to predict whether Left Ventricular Systolic Dysfunction (LVSD) is causative for this symptom. The breathing not properly data and studies by Morrison, et al. demonstrated a high positive predictive value (PPV) for predicting congestive heart failure as the etiology of patients presenting with dyspnea to the emergency department [1–5]. The sensitivity and specificity of the BNP assay has been reported to be high, if serum concentrations are greater than 100 picograms per milliliter (pg/mL) for BNP and 500 pg/mL for NT-ProBNP for predicting LVSD as the etiology of dyspnea [6, 7]. Thus with a high pretest probability, members of the emergency department utilize the NT-ProBNP and BNP in the decision tree to treat patients presenting with dyspnea. The presence of chronic kidney disease (CKD) has been recognized to alter their predictive value and several authors have suggested higher cut-off points for various degrees of renal impairment [8–16].

We could not identify any literature that validates the use of NT-pro BNP in patients with acute onset of dyspnea and advanced CKD or end-stage renal disease (ESRD). Our study retrospectively reviewed the charts of 250 consecutive admissions where and ESRD patient presented with dyspnea. We conclude that this diagnostic purpose NT-ProBNP plays no role in the management of hemodialysis patients.

## 2. Materials and Methods

### 2.1. Study Design

This study was approved by the Cooper University Hospital Institutional Review Board and was in compliance with the regulations set forth by the Declaration of Helsinki. It was designed as a retrospective case series of 250 subjects with medical record abstraction beginning from March 31, 2011 and working backward in time to July, 2010. Eligible patients 18 years or older were admitted to Cooper University Hospital with the diagnosis code of ICD-9 = 585.6 or end-stage renal disease on hemodialysis (ESRD on HD), presenting with acute symptoms of dyspnea, shortness of breath, or respiratory distress. This excluded patients without end-stage renal failure and subjects not on HD. We also excluded subjects who presented with complaints other than shortness of breath, chronic respiratory complaints, subjects under 18, and undergoing peritoneal dialysis or acute hemodialysis.

The sample was described in terms of age (years), sex, race, weight prior to dialysis and estimated dry weight (kg), creatinine (mg/dL), NT-ProBNP (pg/mL), hemodialysis was within 24 hours of admission (yes/no) and volume removed (L), cardiology consultation placed (y/n), echocardiogram performed (y/n) and estimated ejection fraction (%), furosemide administration (y/n), infectious disease consultation placed (y/n) and administration of antibiotics (y/n).

For purposes of analysis patients were categorized into one of three groups: NT-ProBNP elevated (elevated test for age), NT-ProBNP normal (test within normal range for age), or no NT-ProBNP (test not performed). NT-ProBNP patients were further categorized as either normal ejection fraction (EF ≥ 45%) or low ejection fraction (<45%).

### 2.2. Study Measurements

Patient subgroups were tested for significant differences on NT-ProBNP, creatinine, weight (pre-/post-HD), and volume fluid removed during HD. Weight was calculated by standardized measurements on scales “zeroed” daily by hospitals quality control staff. Volume removed was extrapolated from the medical record by nephrology attending physician using notes on the actual amount of postdialysis fluid removed from the patient. Serum creatinine was measured by spot venous 5 sampling, centrifugation, storage at −20 degrees Celsius and then analyzed via Isotope Dilution Mass Spectrometry (IDMS) or standard measurements. NT-ProBNP samples were collected by similar means as the creatinine and measured by electro-chemiluminescence immunoassay. Again both laboratory-testing devices are “zeroed” daily by laboratory staff.

Cardiology staff estimated ejection fraction primarily by transthoracic echocardiography (TTE). M-mode two-dimensional imaging and spectral/color flow Doppler recording were obtained. Measurement we performed using short-axis, parasternal long axis and apical 2 and 4 chamber views. Measurements were performed by guidelines set forth by the American Society of Echocardiography using the Quinones formula for the parasternal long-axis, Simpson method in the 2 and 4 chamber views [17, 18]. Parameters gathered included Left Ventricular Ejection Fraction (LVEF), Right Ventricular Ejection Fraction (RVEF), quantification of mitral regurgitation, quantification of tricuspid regurgitation, estimated systolic pulmonary artery pressure, left atrial volume, left ventricular mass, and E/E′.

### 2.3. Statistical Analysis

Differences between subgroups and categorical variables were tested for significance by Pearson X2 or Fishers exact test. Mean differences between subgroups were tested for significance by independent samples *t*-tests. Overall or group specific standard deviations were used depending on Levene's test for homogeneity of variances. Associations between continuous measures were calculated using the Pearson correlation coefficient, with effect size defined as the coefficient of determination.

## 3. Results

### 3.1. Subgroup Analysis

**Figure 1:**
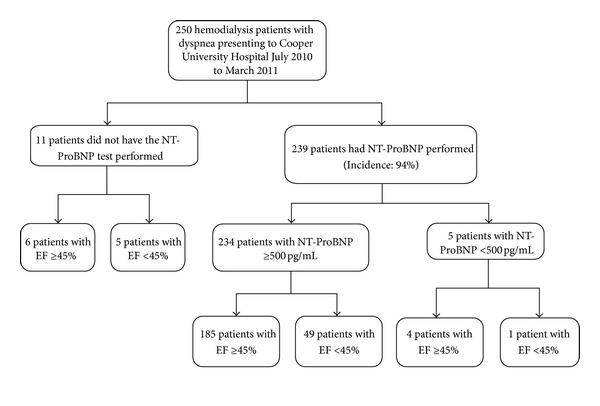
Subject selection of general population.

As seen in Table 1, the study population did not differ in age, sex, or weight. When further examining the study population disbursement, Figure 1 shows that 95.6% of patients had a NT-ProBNP test performed, of whom 97.9% were elevated. The mean values, standard deviations of the no NT-ProBNP, NT-ProBNP elevated, and normal subgroups can be summarized in Table 2.

Table 2 shows that no significant difference was observed between the NT-ProBNP elevated versus normal subgroups or the NT-ProBNP normal and no NT-ProBNP subgroups on age, predialysis weight, estimated dry weight, and volume removed. Similar findings were detected for the predialysis weight, estimated dry weight, and volume removed (*P* = 0.49, and *P* = 0.42, *P* = 0.48, resp.,) of the NT-ProBNP elevated subgroup versus the no NT-ProBNP subgroup. However there was a significant difference among the ages of these groups (*P* = 0.01). 

### 3.2. Correlation Coefficients of NT-ProBNP

Correlation coefficients were calculated to examine correlation between the NT-ProBNP test and mean age, EDW, amount of fluid removed, and EF. Linear regression and slope calculation yielded no correlation between the NT-ProBNP test and age (*r* = 0.05), EDW (*r* = −0.09), amount of volume removed (*r* = 0.07), and EF (*r* = 0.02). 

### 3.3. Incidence of Cardiology Parameters among Subgroups

Amid the cardiology parameters 165 or 55.2% of NT-ProBNP elevated subjects received a cardiology consult, and 58.7% of subjects received an echocardiogram with a mean EF of 54.6%. This is comparable to 50% of the NT-ProBNP normal group and 45.5% of the no NT-ProBNP group receiving both cardiology consult and echocardiography. The ejection fractions of these groups are 50.8% and 45.5% showing no significant difference when compared with the EF of the elevated subgroup (*P* = 0.43 and 0.55, resp.).

 Similar symmetry was detected when measuring other echocardiography parameters. The Mean RVEFs were 38.2%, 37.5%, and 40.9% for the NT-ProBNP elevated, normal, and no NT-ProBNP groups, respectively. The mean mitral valve areas (MVA) were 4.2 cm^2^, 4.6 cm^2^, and 4.5 cm^2^, respectively. The mean mitral valve gradients were 3.4 mmHg, 4.2 mmHg, and 3.7 mmHg. The tricuspid valve parameters yielded a mean jet area-central jets at under 5 cm^2^ for each group. The mean vena contracta width was not defined in each group, indicating normal values. The mean pulmonary artery systolic pressures of the NT-ProBNP elevated, normal, and no BNP groups were 20.1 mmHg, 22.4 mm Hg and 19.7 mmHg respectively.

The mean left atrial volumes were 47.3, 45.5, and 39.2 mL, respectively. The mean Left ventricular masses for the elevated, normal, and no NT-ProBNP groups were 148.8 grams (g), 139.2 g, and 156.7 g, respectively. Lastly, the E/E' ratio were all above 15 in each group reported. A summary of these results can be seen in Table 4.

### 3.4. Subanalysis of Extreme Groups

**Figure 2:**
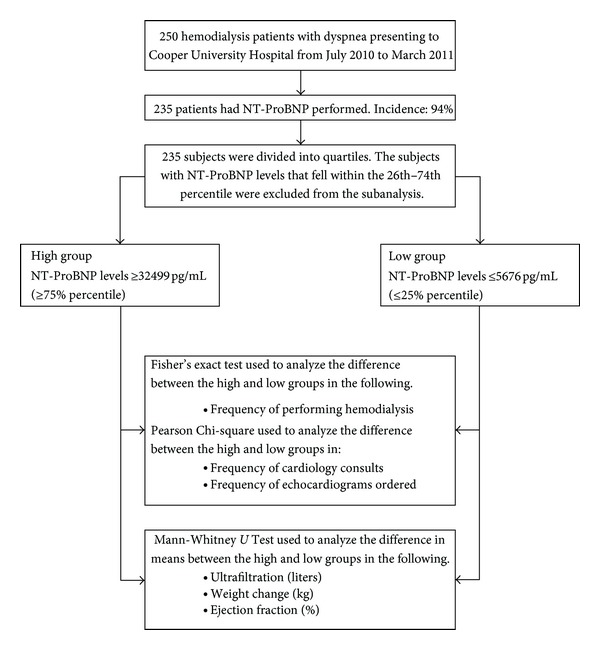
Subject selection of extreme quartiles.

Using the data collected, an extreme group of patients were identified. The 239 patients with NT-ProBNP levels were divided into quartiles. The lower quartile (≤25%) was labeled as the “low group” and had levels, which corresponded to 5676 pg/mL and below. The upper quartile (≥75%) was labeled as the “high group” and had NT-ProBNP levels which corresponded to 32499 pg/mL and above. There were 60 subjects in the low group and 61 subjects in the high group (Figure 2). 

Analysis between the two groups to assess the difference in the frequency of performing hemodialysis was done using a Fisher's exact test. Fifty-five out of 59 (93.2%) subjects in the low group underwent hemodialysis while all 61 (100%) subjects in the high group had hemodialysis. There was a marginal statistical difference between the two groups (*P* < 0.05).

A Pearson Chi-Square analysis was performed between the low and high groups to assess the difference in the frequency of ordering cardiology consults and echocardiograms. Cardiology consults were ordered in 33 out of 58 (56.9%) subjects in the low group while 47 out of 61 (77%) subjects in the high group had cardiology consults. There was a significant difference between both groups (*P* = 0.02). In the low group, 29 out of 60 (48.3%) subjects had echocardiograms ordered compared to 39 out of 61 (63.9%) from the high group. There was no significant difference between the two groups in ordering echocardiograms (*P* = 0.08).

A Mann-Whitney *U* Test evaluated the difference in volume of fluid removal, weight change, and ejection fraction between low and high groups (Table 3). The mean ultrafiltration for the low group was 2.11 liters (*n* = 59) compared to 2.48 liters for the high group (*n* = 61). The mean weight change was 2.29 kg for the low group (*n* = 59) and was 2.70 kg for the high group (*n* = 61). Low group (*n* = 60) ejection fraction mean was 54.2% compared to 50.66% for the high group (*n* = 61). There was no statistically significant difference between the low and high groups in terms of volume of fluid removal (*P* = 0.14) and weight change (*P* = 0.22). There was a statistically significant difference in ejection fraction (*P* = 0.02).

## 4. Discussion

To examine the utility of the NT-ProBNP test in the setting of acute dyspnea for subjects with ESRD on HD, one must first understand the test's molecular structure. Brain Natriuretic Peptide is a 32-amino acid polypeptide secreted by the ventricles in response to stretch [19–21]. Similarly, a 76-amino acid N-terminus fragment of the BNP (NT-ProBNP) hormone is also secreted as the inactive component. The NT-ProBNP molecule is a cleaved molecule from ProBNP, which is exclusively secreted by the kidneys via endocytosis [20, 21]. In the setting of normal renal function, BNP has a 20-minute half-life and for NT-BNP 1 to 2 hours [10–13]. It is for this reason that we chose the NT-ProBNP test, instead of the BNP test, to determine LVSD in patients with ESRD on HD, who presented to the emergency department with acute dyspnea.

 Both of these hormones have been used in patients presenting with a chief complaint of dyspnea, to predict whether Left Ventricular Systolic Dysfunction (LVSD) is the cause for their symptoms. The Breathing Not Properly data and studies by Morrison et al. demonstrated a positive predictive value (PPV) of greater than 90% for predicting congestive heart failure as the etiology of patients presenting with dyspnea to the emergency department [1–5]. The sensitivity of the BNP assay has been reported to be 90% and specificity of 76% if serum concentrations are greater than 100 picograms per milliliter(pg/mL) for BNP and 500 pg/mL for NT-ProBNP for predicting CHF in patients presenting with dyspnea [6, 7]. Thus with a high pretest probability, members of the emergency department utilize the NT-ProBNP and BNP to treat patients presenting with dyspnea. 

This ability has prompted diagnostic algorithms in emergency departments across the country for utilizing the NT-ProBNP as a marker for determining the etiology of people presenting with shortness of breath. However, in circumstance of renal dysfunction, the diagnostic ability of NT-ProBNP test may be less apparent [14–16]. It has been clearly demonstrated that the BNP testing has prognostic ability in patients with ESRD [22–26]. Despite the prognostic ability of the BNP test, clinicians lack clear data on the diagnostic utility of this test in the setting of ESRD on HD. 

Our study demonstrated that 95.6% of the sample population received a NT-ProBNP test among which 98% was determined to be an “elevated” test. Following the diagnostic algorithm, an elevated NT-ProBNP would likely correlate with ordering of an echocardiogram or cardiology to assess LV systolic function (LVSF). Although the test was shown by Satyan et al. to correlate with LV dysfunction, we could not find any clinical value in determining disposition of emergency room patients [27]. Yet despite this, similar percentages of the study population received both cardiology consultation and echocardiography even when comparing the NT-ProBNP elevated group with the NT-ProBNP normal and the group in which no test was performed (Table 2).

Using a similar logic, there were no significant differences found between the predialysis weights, EDW, volumes removed, or EFs when comparing the NT-ProBNP elevated with the NT-ProBNP normal and the group in which no test was performed (Table 2). To further illustrate this point, echocardiographic parameters were examined. Across each group, there were no significant differences found between these parameters as exemplified in Table 4. All of this data points away from the diagnostic utility of the NT-ProBNP test in this setting.

In the extreme cases of low versus high NT-ProBNP levels, there was a marginally statistical difference in management as evidenced by a higher incidence of performing hemodialysis and ordering cardiology consults in the group with the upper quartile NT-ProBNP. There was, however, no significant difference found in the volume of fluid removal and the weight change between pre- and postdialysis (Table 3).

 Potential limitations of this study included the lack of data on subjects receiving a BNP test. Another potential limitation is that data was gathered retrospectively. This study also does not take into account individual cases, but rather trends in our sample population. Attempts to counteract these potential limitations will be made through future prospective studies to examine the utility of the NT-ProBNP test as well as BNP test.

## 5. Conclusion

In patients with ESRD on HD who presented to the ED with acute dyspnea there is no significant difference in the incidence of cardiology/infectious disease consultation, furosemide/antibiotics administration. Additionally no difference was seen in the incidence of neither echocardiography ordered nor the parameters measured by standard echocardiography means.

## Figures and Tables

**Table 1:** Sample population demographics.

	NT-ProBNP		No NT-ProBNP	Total (*n* = 250)
	NT-ProBNP elevated	NT-ProBNP normal		
Mean age^1^	58.3	51.8	46.6	57.7
Sex				
Males	108	2	2	112
Females	127	2	9	138
Race				
Black	98	1	3	102
White	65	0	6	71
Hispanic	71	3	2	76
Asian	1	0	0	1
Other	0	0	0	0

^1^Units = years.

**Table 2:** Mean, median, and standard deviation of subgroups via systolic function.

NT-ProBNP group	EF (%)			Creatinine^1^	HD performed	Volume removed^2^	Weight prior to dialysis^3^	Weight after dialysis^3^	Cardiology consult	ECHO	Furosemide	Infectious disease consult	Antibiotics
No NT-ProBNP	<45	*N*	Valid	6	6	6	6	6	6	6	6	6	6
			Mean	8.95	1.00	2.43	72.57	68.77	0.00	0.00	0.00	0.33	0.33
			Median	9.90	1.00	2.00	67.70	65.00	0.00	0.00	0.00	0.00	0.00
			Std. deviation	3.91	0.00	1.40	10.50	10.13	0.00	0.00	0.00	0.52	0.52
	≥45	*N*	Valid	5	5	5	5	5	5	5	5	5	5
			Mean	8.00	1.00	2.00	77.20	75.68	1.00	1.00	0.20	0.20	0.20
			Median	8.10	1.00	2.00	74.30	68.65	1.00	1.00	0.00	0.00	0.00
			Std. deviation	2.39	0.00	0.00	14.34	18.08	0.00	0.00	0.45	0.45	0.45

NT-ProBNP normal	<45	*N*	Valid	1	1	1	1	1	1	1	1	1	1
			Mean	10.45	0.00	0.00	136.34	136.34	1.00	0.00	0.00	0.00	0.00
			Median	10.45	0.00	0.00	136.34	136.34	1.00	0.00	0.00	0.00	0.00
	≥45	*N*	Valid	4	4	4	4	4	4	4	4	4	4
			Mean	9.23	0.75	2.28	78.73	77.78	0.50	0.75	0.00	0.25	0.25
			Median	6.60	1.00	2.55	72.50	71.35	0.50	1.00	0.00	0.00	0.00
			Std. deviation	8.52	0.50	1.72	14.66	14.42	0.58	0.50	0.00	0.50	0.50

NT-ProBNP elevated	<45	*N*	Valid	49	49	49	49	49	49	49	48	49	49
			Mean	6.00	0.98	2.45	72.36	71.60	0.59	0.49	1.60	0.12	0.14
			Median	5.60	1.00	2.00	72.50	70.38	1.00	0.00	0.00	0.00	0.00
			Std. deviation	2.31	0.14	1.14	20.97	19.28	0.50	0.51	6.55	0.33	0.35
	≥45	*N*	Valid	185	185	185	185	185	185	185	185	185	185
			Mean	6.69	0.98	2.44	81.88	79.10	0.74	0.61	0.72	0.09	0.12
			Median	6.10	1.00	2.00	75.90	72.50	1.00	1.00	0.00	0.00	0.00
			Std. deviation	3.28	0.15	1.13	24.96	24.69	0.44	0.49	5.76	0.28	0.33

^1^Creatinine in milligrams/deciliter (mg/dL), ^2^hemodialysis performed in liters, and ^3^weight in kilograms (kg).

**Table 3:** Comparison of the high NT-ProBNP and low NT-ProBNP quartiles^1^ using the Mann-Whitney *U * Test.

	Group	*N*	Mean	Standard deviation	*P* value
Ultrafiltration (liters)	Low	59	2.11	1.18	0.14
	High	61	2.48	1.16	

Weight change (kg)	Low	59	2.29	1.65	0.22
	High	61	2.70	1.66	

Ejection Fraction (%)	Low	60	54.20	18.22	0.02
	High	61	50.66	15.91	

^1^Low group NT-ProBNP levels ≤5676 pg/mL (≤25% percentile).

High group NT-ProBNP levels ≥32499 pg/mL (≥75% percentile).

**Table 4:** The Echocardiography parameters of the NT-ProBNP elevated, NT-ProBNP normal, and no NT-ProBNP groups.

	NT-ProBNP		No NT-ProBNP	*P* Value (95% CI)
	NT-ProBNP elevated	NT-ProBNP normal		
Left ventricle parameters				
Left ventricular systolic ejection fraction (%)^1^	54.6%	50%	45.5%	0.43
Left atrial volume (mL)^2^	47.3	45.5	39.2	0.66
Left ventricular mass (g)^3^	148.8	139.2	156.7	0.45
E/E′ ratio	>15	>15	>15	1.0
Tricuspid parameters				
Jet area-central jets (cm^2^)^4^	<5	<5	<5	1.0
Vena contracta width	Not defined	Not defined	Not defined	N/A
Mitral valve parameters				
Mitral valve areas (cm^2^)^4^	4.2	4.6	4.5	0.87
Mitral valve gradient (mmHg)^5^	3.4	4.2	3.7	0.63
Right ventricular parameters				
Right ventricular ejection fraction (%)^1^	38.2%	37.5%	40.9%	0.46
Pulmonary artery parameters				
Pulmonary artery systolic pressures (mmHg)^5^	20.1	22.4	19.7	0.24

^1^Percent (%), ^2^mililiters (mL), ^3^grams (g), ^4^centimeters squared (cm^2^), and ^5^milimeters of Mercury (mmHg).
